# The molecular classification of astrocytic tumors

**DOI:** 10.18632/oncotarget.22047

**Published:** 2017-10-25

**Authors:** Chen-Xue Mao, Ji-Ye Yin, Ying Zhang, Zhi-Bin Wang, Zhi-Quan Yang, Zheng-Wen He, Xiang-Min Li, Xiao-Yuan Mao, Ru-Tao Cui, Xue-Jun Li, Xi Li, Wei Zhang, Hong-Hao Zhou, Zhao-Qian Liu

**Affiliations:** ^1^ Department of Clinical Pharmacology, Xiangya Hospital, Central South University, Changsha 410008, P. R. China; ^2^ Department Neurosurgery, Xiangya Hospital, Central South University, Changsha 410008, P. R. China; ^3^ Institute of Clinical Pharmacology, Central South University, Hunan Key Laboratory of Pharmacogenetics, Changsha 410078, P. R. China; ^4^ Department of Neurosurgery, The Affiliated Cancer Hospital of Xiangya School of Medicine, Central South University, Changsha 410014, P. R. China; ^5^ Department of Emergency, Xiangya Hospital, Central South University, Changsha 410008, P. R. China; ^6^ Departments of Pharmacology and Experimental Therapeutics, Boston University School of Medicine, Boston, MA 02118, USA

**Keywords:** astrocytoma, calcium signaling pathway, IDH, molecular classification, chemokine signaling pathway

## Abstract

**Aim:**

This study will explore the genetic and epigenetic alterations in astrocytomas, and identify the critical molecular signatures and signaling pathways for prognosis assessment by multiplatform comprehensive analysis.

**Method:**

We performed integration analyses of incorporating DNA methylation, mRNA expression, microRNA expression, and long non-coding RNA (lncRNA) expression in 33 astrocytic tumor tissues and 9 non-tumor brain tissues.

**Result:**

We observed that 11,795 DNA methylation sites, 3,627 genes, 136 microRNAs, and 3,334 lncRNAs were significantly differential between tumors and non-tumor brain tissues, and the filtered signatures through comprehensive analysis were significantly enriched in calcium signaling pathway. Furthermore, four signatures involved in calcium signaling pathway and age could contribute to predicting the patients’ overall survival. Additionally, we identified differentially expressed signatures between IDH-mutated and IDH wild-type astrocytic tumors, and complement and coagulation cascades pathway was the most significant pathway in functional enrichment analysis using multiplatform data. The IDH wild-type astrocytomas were divided into two subtypes by Cluster of Cluster (CoC) analysis, one of which was enriched for astrocytomas overexpressed in chemokine signaling pathway.

**Conclusion:**

The calcium signaling pathway played a key role in astrocytoma tumorigenesis and prognosis. IDH mutation was a vital biomarker, and resulted in the change of expression level in complement and coagulation cascades pathway. The chemokine signaling pathway could characterize subtypes of IDH wild-type astrocytomas.

## INTRODUCTION

Among primary central nervous system tumors, gliomas are the largest and most diverse group [1]. Gliomas can be classified as astrocytomas, oligodendrogliomas or oligoastrocytomas and graded as I to IV according to the histopathological classification of World Health Organization [2]. Glioblastoma (GBM), the grade IV astrocytoma, arises most frequently and has the worst prognosis with median survival of 1 year [3]. The widely infiltrative properties make astrocytoma impossible to be completely resected and likely to malignantly progress and recur, resulting in highly variable survival periods of patients. The single traditional diagnostic method based on histopathologic characteristics cannot satisfy the requirement of predicting clinical outcomes [4–6].

More recently, understanding of genetic biomarker has been improved along with the discovery of numerous molecular alterations in gliomas. Some molecular alterations are now recognized as major biomarkers of clinical behavior and molecular classification, including IDH mutation, 1p/19q codeletion, TERT mutation and MGMT methylation [7–13]. However, the genetic and epigenetic characteristics of astrocytomas have not been extensively explored by multiplatform comprehensive analysis.

In the present study, we investigated the DNA methylation and the expression level of genes, miRNAs, and long non-coding RNAs (lncRNAs) in 33 astrocytic tumor tissues and 9 non-tumor brain tissues by microarray analysis, predicted the potential biological function of significantly differentially expressed signatures by integration analysis of multiplatform data, and assessed the prognostic value. We also observed the effect of IDH mutation on DNA methylation and gene expression level. Finally, we constructed a molecular classification of IDH wild-type astrocytomas and identified the biomarker of classification.

## RESULTS

### Clinical characteristics of the astrocytoma cases

We found IDH mutations in 30.30% of astrocytomas samples and 51.51% for TERT mutations, with one sample having both mutations. Differences in the prevalence of IDH mutation and MGMT promoter methylation between lower-grade astrocytomas and grade IV GBM were significant. Nearly all astrocytomas with IDH mutation were lower-grade astrocytomas, and the prevalence of MGMT promoter methylation in lower-grade astrocytomas was higher than that in GBM (p=0.032, Fisher's exact test). Samples of astrocytomas with MGMT promoter methylation had less TERT mutations than samples with low MGMT promoter methylation (p=0.041). Previous studies showed that 1p/19q codeletion occurred most often in oligodendroglial gliomas. We identified only 5 astrocytomas with 1p/19q codeletion in this study, and they all were IDH wild-type and MGMT promoter methylation samples. The clinical information of patients is provided in Table 1.

**Table 1:** Clinical characteristics of patients

Characteristic	Classes	Tumor	Non-tumor
Age	≤50	22 (66.67%)	3(33.33%)
	>50	11 (33.33%)	6(66.67%)
Gender	Male	20 (60.61%)	7(77.78%)
	Female	13 (39.39%)	2(22.22%)
IDH mutation	wild type	23 (69.70%)	
	mutation	10 (30.30%)	
TERT promoter mutation	wild type	14 (42.42%)	
	mutation	17 (51.51%)	
	absent	2 (6.06%)	
1p/19q codeletion	no codeletion	28 (84.85%)	
	codeletion	5 (15.15%)	
Grade	II-III	17 (51.52%)	
	IV	16 (48.48%)	
MGMT methylation	Low	12 (36.36%)	
	High	21 (63.64%)	

### Identification of differential signatures between astrocytic tumors and non-tumor brain tissues

First, a total of 11,795 probes were identified as differentially methylated by the criteria of corrected p-value <0.05 and absolute β-difference >0.2 between 33 astrocytic tumors and 9 non-tumor brain tissues (Supplementary Figure 1B). Then, the two-dimensional hierarchical clustering was performed using the top 5000 differentially methylated probes between tumors and NTL tissues (Supplementary Figure 2B). Finally, we tested 6 DNA methylation probes using pyrosequencing in 65 astrocytic tumors and 16 non-tumor brain tissues. The results demonstrated that 4 probes were significantly hypermethylated and 2 probes were significantly hypomethylated in astrocytic tumors (Supplementary Figure 3), which is consistent with microarray results.

Moreover, we identified 3,627 genes, 3,334 lncRNAs and 136 miRNAs differentially expressed by the criteria of corrected p-value <0.05 and fold change ≥2 in 33 astrocytic tumors and 9 non-tumor brain tissues (Supplementary Figure 1A, 1C and 1D). The two-dimensional hierarchical clustering was performed using the differentially expressed genes, lncRNAs and miRNAs, respectively (Supplementary Figure 2A, 2C and 2D). All heatmaps revealed clear separation of tumors and NTL tissues. The expression of 10 genes, 2 miRNAs and 6 lncRNAs in astrocytic tumors and non-tumor brain tissues were validated by quantitative real-time PCR (Supplementary Figures 4-6), and the results coincided with that of microarrays.

**Figure 1:**
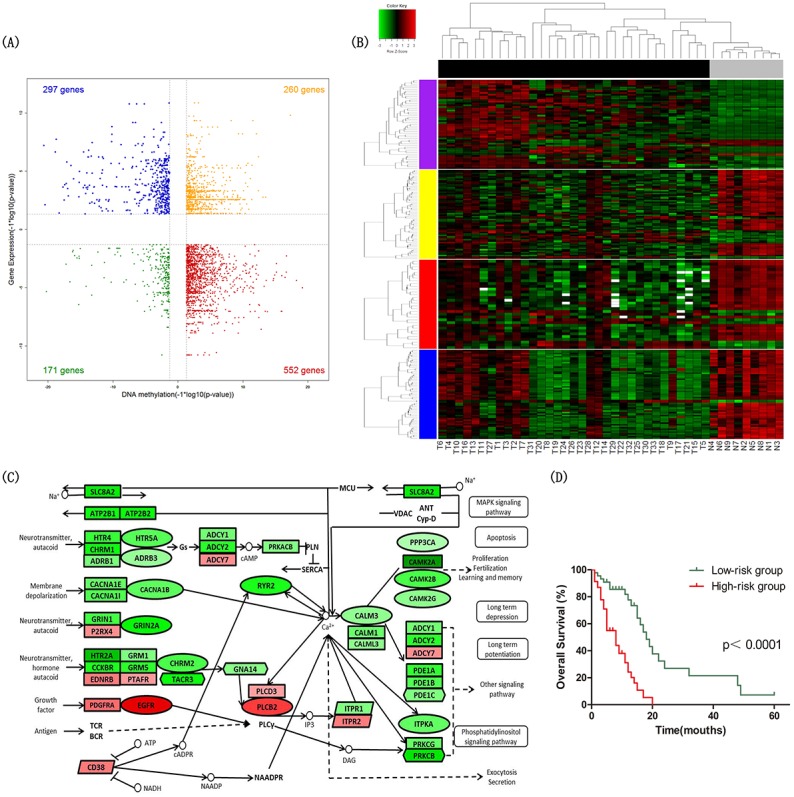
Identification of differential signatures between astrocytic tumors and non-tumor brain tissues **(A)** Starburst plot integrating differential DNA methylation and gene expression analyses. Indicated are genes that are hypermethylated and down-regulated in tumors (red); hypomethylated and up-regulated in tumors (blue); hypermethylated and up-regulated in tumors (yellow); or hypomethylated and down-regulated in tumors (green). **(B)** Two-dimensional hierarchical clustering of the significantly differential signatures in calcium signaling pathway in all samples (33 tumor tissues in black vs. 9 NTL tissues in gray). Signatures are in rows (51 differentially methylated and gene expression direction inversed probes in purple; 62 differentially expressed genes in yellow; 32 differentially expressed miRNAs in red; 59 differentially expressed lncRNAs in blue); samples are in columns. **(C)** Schematic representation of calcium signaling pathway. Ellipses represent genes overlapped in differential signatures of four platforms; rectangles represent genes within miRNA-gene co-expression pairs; parallelograms represent genes within lncRNA-gene co-expression pairs but not within miRNA-gene co-expression pairs; hexagons represent genes significantly hypermethylated and down-regulated or significantly hypomethylated and up-regulated, but not within miRNA-gene co-expression pairs. Gene color corresponds to fold change (genes colored in red are up-regulated, genes colored in green are down-regulated). **(D)** Kaplan–Meier curves of overall survival in astrocytoma patients. Patients were classified into high-/low-risk group according to the risk score formula combining signatures involved in calcium signaling pathway and age.

By integrating the gene expression profiles and DNA methylation profiles, 552 genes were statistically significantly hypermethylated and down-regulated, while 297 genes were significantly hypomethylated and up-regulated (Figure 1A). We identified 196 hypermethylated and down-regulated genes showing strongly inverse correlation between DNA methylation and gene expression by calculating the Pearson correlation coefficients (PCC) (PCC < -0.5). Among these genes, ANK3 was ranked highest based on the PCC of three methylation sites ranging from -0.75 to -0.87 (cg22150335, cg20135031 and cg14391247) (Supplementary Figure 7A-7C). In addition, TSPYL5, RAB3A, ABCA2, NCDN and DLGAP3 were also noteworthy. Scatterplots of these genes expression versus DNA methylation are shown in (Supplementary Figure 7D-7L).

Potential gene targets for each dysregulated miRNA were obtained by miRWalk (http://www.umm.uniheidelberg.de/apps/zmf/mirwalk), which combined prediction results of different algorithms including DIANAmT, miRanda, miRDB, miRWalk, RNAhybrid, PICTAR4, PICTAR5, PITA, RNA22 and Targetscan. The predicted gene targets were specifically identified by at least 3 of these programs. Then, the Pearson correlation coefficients (PCC) between expression of the miRNA and their target genes were calculated. Finally, we identified 6,716 miRNA-gene co-expression pairs composed of 103 miRNAs and 1,645 genes with PCC <-0.7 or > 0.7 and p-value <0.05. In addition, for each dysregulated lncRNA, the Pearson correlation coefficients (PCC) of its expression and that of each dysregulated gene was calculated. 70,948 lncRNA-gene co-expression pairs composed of 2,419 lncRNAs and 2,848 genes were found by PCC < -0.8 or > 0.8 and p-value < 0.05. Taken together, 315 genes overlapped in integration analyses were selected for further analysis.

The Gene Ontology (GO) and Kyoto Encyclopedia of Genes and Genomes (KEGG) pathway analysis was performed to determine the biological functions of these 315 genes by DAVID database (http://david.abcc.ncifcrf.gov/). The set of 315 genes was significantly enriched in KEGG pathways including calcium signaling pathway (Benjamini-Hochberg adjusted p-value=0.029) and neuroactive ligand-receptor interaction (Benjamini-Hochberg adjusted p-value=0.042) (Table 2). The findings suggested that calcium signaling pathway played a key role in astrocytoma, and the genes of calcium signaling pathway in the 315 gene set were listed in Supplementary Table 4. The hierarchical clustering analysis of the significantly differential signatures involved in calcium signaling pathway was shown in Figure 1A.

**Table 2:** The top significantly enriched KEGG pathways in integration analyses of tumor vs. non-tumor tissues

KEGG pathway	p-value	BH adjusted p-value
Calcium signaling pathway	2.388×10^-4^	0.029
Neuroactive ligand-receptor interaction	7.003×10^-4^	0.042
Glioma	9.285×10^-3^	0.316
Long-term potentiation	0.013	0.322
ErbB signaling pathway	0.033	0.561
Prion diseases	0.035	0.512

### Survival prediction analysis by the signatures of calcium signaling pathway

To validate multiplatform differentially expressed signatures involved in calcium signaling pathway were significantly associated with overall survival of astrocytoma patients, we did univariate Cox regression analysis using multiplatform data of 92 GBM samples in TCGA.

We identified 13 DNA methylation probes, 2 genes, 2 miRNAs involved in calcium signaling pathway and age that were independently associated with overall survival. After adjusted multivariate Cox regression analysis for the age, 9 DNA methylation probes, 1 gene, 2 miRNAs were significantly correlated with overall survival. To establish a prediction model, multivariable Cox regression analysis was performed using a backward stepwise method, and we selected CHRM2 cg02866106, CACNA1B cg05863502, CALM1 cg18079499, miR-183 and age to construct the prognostic model. We established a formula comprised of the 4 signatures (correlated with good prognosis) and age (correlated with poor prognosis) to calculate the risk score for every patient. Risk score = (0.055 × age) – (1.517 × beta value of CHRM2 cg02866106) – (1.398 × beta value of CACNA1B cg05863502) – (5.060 ×beta value of CALM1 cg18079499) - (0.584 × expression value of miR-183). By the cutoff of median risk score (-3.058), patients were divided into high-risk or low-risk groups according to the value of risk score. Kaplan–Meier analysis showed that patients in high-risk group had significantly shorter overall survival than patients in low-risk group (median survival 8 months vs 18 months, p<1×10^-4^) (Figure 1D).

### Identification of differential signatures between IDH-mutated and IDH wild-type astrocytic tumors

To identify subtypes and the vital biomarker of astrocytomas, we unsupervised clustered multiplatform data on 33 astrocytoma tumors.

The median absolute deviation (MAD) was calculated for each DNA methylation probe and the top 3,000 probes were selected for non-negative matrix factorization (NMF) clustering analysis. The NMF clustering and rank estimates were calculated for ranks 2-5 with default settings of method brunet and seed random. A two-cluster result was determined by rank survey profiles for cophenetic coefficient and visual inspection of the correlation matrices (Supplementary Figure 8B). By the same method, the 3,000 genes, 150 miRNAs and 1,500 lncRNAs with the highest median absolute deviation (MAD) were defined as three clusters, respectively (Supplementary Figure 8A, 8C and 8D). In all unsupervised clustering analysis results, one of the clusters was closely related to IDH mutation. The consistent results indicated that IDH mutation could define a subtype of astrocytoma.

**Figure 2:**
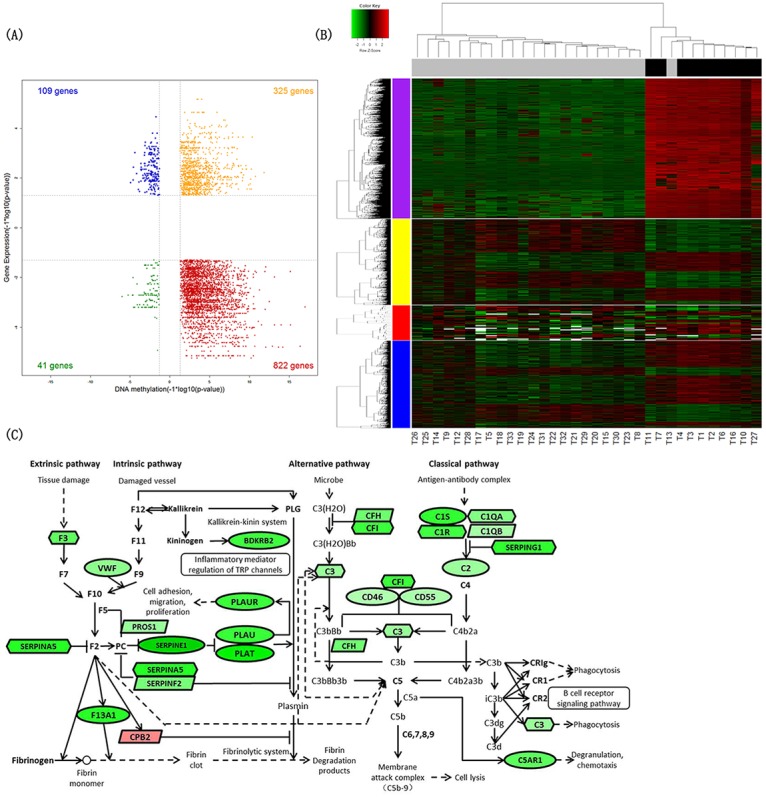
Identification of differential signatures between IDH-mutated and IDH wild-type astrocytic tumors **(A)** Starburst plot integrating differential DNA methylation and gene expression analyses. Indicated are genes that are hypermethylated and down-regulated in IDH-mutated tumors (red); hypomethylated and up-regulated in IDH-mutated tumors (blue); hypermethylated and up-regulated in IDH-mutated tumors (yellow); or hypomethylated and down-regulated in IDH-mutated tumors (green). **(B)** Two-dimensional hierarchical clustering of the significantly differential signatures in IDH-mutated astrocytic tumors (10 IDH-mutated astrocytic tissues in black vs. 23 IDH wild-type astrocytic tissues in gray). Signatures are in rows (top 5,000 differentially methylated probes in purple; 1,862 differentially expressed genes in yellow; 42 differentially expressed miRNAs in red; 1,734 differentially expressed lncRNAs in blue); samples are in columns. **(C)** Schematic representation of complement and coagulation cascades pathway. Ellipses represent genes overlapped in differential signatures of four platforms; rectangles represent genes within miRNA-gene co-expression pairs; parallelograms represent genes within lncRNA-gene co-expression pairs but not within miRNA-gene co-expression pairs; hexagons represent genes significantly hypermethylated and down-regulated or significantly hypomethylated and up-regulated, but not within miRNA-gene co-expression pairs. Gene color corresponds to fold change (genes colored in red are up-regulated, genes colored in green are down-regulated).

To determine the effect of IDH mutation on DNA methylation and RNA expression, we did a differential analysis between 10 IDH-mutated and 23 IDH wild-type astrocytic tumors, and 28,609 DNA methylation probes, 1,862 genes, 42 miRNAs and 1,734 lncRNAs were identified differentially expressed in total. These differentially expressed signatures were used to perform hierarchical clustering analysis respectively, and all the heatmaps showed good separation of IDH-mutated and IDH wild-type astrocytic tumors in general (Figure 2B).

By the integration analysis of multiplatform data as described above, we found the signatures involving complement and coagulation cascades pathway significantly changed in IDH-mutated astrocytomas (Benjamini-Hochberg adjusted p-value=2.417×10^-6^) (Table 3). The differentially expressed genes between 6 IDH-mutated and 83 IDH wild-type GBM were also significantly associated with complement and coagulation cascades pathway in TCGA dataset (Benjamini-Hochberg adjusted p-value=0.010) (Supplementary Table 5).

**Table 3:** The top significantly enriched KEGG pathways in integration analyses of IDH-mutated vs. IDH wild-type tumors

KEGG pathway	p-value	BH adjusted p-value
Complement and coagulation cascades	2.259×10^-8^	2.417×10^-6^
ECM-receptor interaction	1.716×10^-6^	9.179×10^-5^
Focal adhesion	9.186×10^-6^	3.276×10^-4^
Cell adhesion molecules (CAMs)	0.029	0.542
p53 signaling pathway	0.032	0.498
Viral myocarditis	0.036	0.483

### The molecular classification of IDH wild-type astrocytomas

To construct molecular classification in IDH wild-type astrocytomas, we calculated the MAD value in multiplatform data and selected the top 3,000 DNA methylation probes, 1,500 genes, 150 miRNAs and 3,000 lncRNAs for NMF analysis, respectively. Unsupervised clustering identified two stable clusters in DNA methylation (M1, M2) (Supplementary Figure 9B), mRNA (m1, m2) (Supplementary Figure 9A), miRNA (mi1, mi2) (Supplementary Figure 9C) and lncRNA (lnc1, lnc2) (Supplementary Figure 9D) expression data from 23 IDH wild-type astrocytoma samples. TERT promoter mutation was predominantly a feature of M1 DNA methylation cluster, 88.89% of samples in M1 cluster harbored a TERT promoter mutation. M1 cluster also was highly enriched for histologic grade IV GBM. 1p/19q codeletion was found in 5 samples, 4 of which were classified in mi2 cluster.

**Figure 3:**
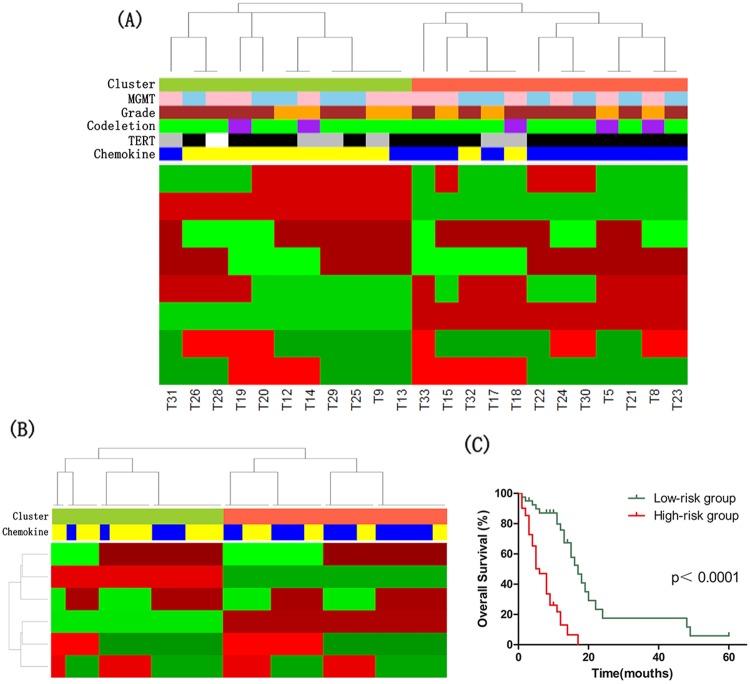
The molecular classification of IDH wild-type astrocytomas **(A)** Cluster of Cluster (CoC) Analysis of IDH wild-type astrocytomas. CoC analysis uses the cluster assignments derived from individual molecular platforms to subdivide tumors, thereby integrating data from analysis of DNA methylation, mRNA, microRNA and lncRNA. For each sample, membership in a particular cluster is indicated by a red tick, and nonmembership is indicated by a green tick. CoC cluster memberships of the tumors are indicated by the color bar: tomato, Cluster CoC 1 (n=12); yellowgreen, Cluster CoC 2 (n=11). Other color bars indicate various molecular features: blue, high expression of chemokine signaling pathway genes (n=12); yellow, low expression of chemokine signaling pathway genes (n=11); black, TERT promoter mutation (n=16); grey, no TERT promoter mutation (n=6); purple, 1p/19q codeletion (n=5); green, no 1p/19q codeletion (n=18); brown, GBMs (n=15); orange, lower-grade astrocytomas (n=8); pink, high MGMT promoter methylation (n=12); skyblue, low MGMT promoter methylation (n=11). White indicates missing value. **(B)** CoC Analyses of IDH wild-type GBM in TCGA dataset. CoC analysis uses the cluster assignments derived from individual molecular platforms to subdivide tumors, thereby integrating data from analysis of DNA methylation, mRNA and microRNA. For each sample, membership in a particular cluster is indicated by a red tick, and nonmembership is indicated by a green tick. CoC cluster memberships of the tumors are indicated by the color bar: tomato, Cluster CoC 1 (n=47); yellowgreen, Cluster CoC 2 (n=36); blue, high expression of chemokine signaling pathway genes (n=41); yellow, low expression of chemokine signaling pathway genes (n=42). **(C)** Kaplan–Meier curves of IDH wild-type astrocytoma patients. Patients were classified into high-/low-risk group based on the risk score formula combining signatures involved in chemokine signaling pathway and age.

Clusters defined from each platform (DNA methylation, mRNA, miRNA, and lncRNA) were formed into a matrix of 1s and 0s. The multiplatform data matrix was unsupervised consensus clustered using the Consensus ClusterPlus R-package (80% sample resampling, 1000 iterations, and Pearson correlation). The robust number of CoC clusters was determined to be 2 using K-means clustering algorithm for clusters ranging 2-7. Based on this, we divided IDH wild-type astrocytomas into two clusters using multiplatform data (Figure 3A).

The significance analysis of microarrays (SAM) method was applied to identify differentially expressed genes of each cluster, and the threshold of statistical significance was q-value<0.1. The highly expressed genes in CoC1 Cluster were significantly enriched in chemokine signaling pathway (Benjamini-Hochberg adjusted p-value=0.012).

Then, we divided 83 GBMs with wild-type IDH in TCGA dataset into two groups by CoC analysis, and found that differentially expressed genes between two clusters were also significantly associated with chemokine signaling pathway (Benjamini-Hochberg adjusted p-value=0.004) (Figure 3B).

To assess the prognostic value of signatures in chemokine signaling pathway, we did univariate Cox regression analysis using DNA methylation, mRNA expression, miRNA expression and survival data of 83 GBM with wild-type IDH in TCGA dataset. The DNA methylation levels of PRKCB cg03217795 and CXCL2 cg23244559, and the expression level of GNG13 were found significantly associated with overall survival. We derived a formula for these signatures and age with the same risk-score method as described above. The risk score of overall survival = (0.054 × age) – (2.155 × beta value of PRKCB cg03217795) – (1.974 × beta value of CXCL2 cg23244559) + (0.359 × expression value of GNG13). The astrocytoma patients with wild-type IDH were respectively classified as low- or high-risk groups of overall survival. Kaplan–Meier analysis showed that patients in low-risk group of overall survival survived significantly longer than patients in high-risk group (median survival 17 months vs 6 months, p<1×10^-4^) (Figure 3C).

## DISCUSSION

Using an integrative, multiplatform approach, we found that calcium signaling pathway played a key role in astrocytoma tumorigenesis, and IDH mutation resulted in the change of gene expression in complement and coagulation cascades pathway. We also determined that chemokine signaling pathway could be the biomarker of molecular classification in IDH wild-type astrocytomas to predict the overall survival.

We identified 11,795 DNA methylation sites, 3,627 genes, 3,334 lncRNAs and 136 miRNAs showing significantly differential in astrocytic tumors. In addition, we found some hypermethylated genes with an associated decrease in expression, such as ANK3 [14, 15], TSPYL5 [16, 17], RAB3A and ABCA2 [18] that have been reported to be related with cancer or central nervous system diseases. Through a comprehensive analysis, DNA methylation, genes, miRNAs and lncRNAs involved in calcium signaling pathway were shown obviously altered in astrocytic tumor tissues compared with non-tumor brain tissues, suggesting that calcium signaling pathway may have critical roles in occurrence and development of astrocytic tumor. Then, the prognostic value of signatures in calcium signaling pathway was verified by risk score method. Astrocytoma patients could be stratified into different tumor risk groups on the basis of risk score combining four signatures and age, and patients in low-risk group survived significantly longer.

Calcium is the second messenger to regulate different cellular functions including cell proliferation and differentiation, development of neural circuits and axonal guidance. Lots of studies have reported that calcium plays a crucial role in the pathogenesis of many central nervous system diseases, such as Alzheimer's disease [19], Huntington's disease [20]. However, reports about the role of calcium signaling pathway in tumor are uncommon. Polisetty et al. found that calcium binding proteins were observably deregulated in GBM [21], indicating that calcium signaling pathway may be a hallmark of astrocytoma, but its specific function and mechanism in tumorigenesis remain to be further investigated. It might provide potential treatment targets and strategies for astrocytoma patients, and its prognostic value could be validated in prospective and multicenter studies. Additionally, neuroactive ligand-receptor interaction pathway has been reported differentially expressed in diffuse intrinsic pontine glioma (DIPG) [22]and Drosophila Parkinson's disease model [23], it also deserves further research.

Consistant with the previous results [24, 25], IDH mutation was a reliable biomarker of astrocytomas in our research by unsupervised clustering analysis of multiplatform data. We also found that it led to significant change of DNA methylation and RNA expression in complement and coagulation cascades pathway. The complement cascade participates in immune response, and previous studies found that complement pathway could supervise and suppress various cancers. Besides, the expression of complement pathway genes correlated with the outcome of AML patients [26]. The coagulation cascade was activated due to the enhanced vasculature permeability in tumor microenvironment, so it may be a potential target for cancer therapy [27]. But the involvement of complement and coagulation cascades pathway in astrocytoma has not been reported.

In the previous studies of The Cancer Genome Atlas (TCGA), the gene expression of GBM were classified as four subtypes proneural, neural, classical and mesenchymal [28]; and the lower-grade gliomas were subdivided into three subtypes: IDH mutation and 1p/19q codeletion; IDH mutation and no 1p/19q codeletion; and IDH wild type using the data of multiple platforms [24]. In addition, Eckel-Passow et al defined five molecular groups in 1,087 gliomas using three markers: IDH mutations, 1p/19q codeletion and TERT promoter mutations [25]. However, the genetic characteristics and molecular classification of biomarkers of astrocytoma remain to be identified, because some alterations are rarely found in astrocytoma, such as 1p/19q codeletion; and there is still lack of molecular classification research in astrocytoma using multiplatform data.

Furthermore, to determine whether IDH wild-type astrocytoma could be subdivided into groups showing distinct behavior, we conducted a classification by CoC analysis. We identified two subtypes of IDH wild-type astrocytoma, one was enriched for astrocytomas with TERT promoter mutation and showed high expression of chemokine signaling pathway genes. Moreover, the overall survival of IDH wild-type astrocytoma patients could be predicted by the risk score formula consisting of 3 signatures involved in chemokine signaling pathway and age.

Existing reports showed that chemokine network could promote the invasiveness and angiogenesis of glioma cells [29]; the chemokine receptor CXCR7 contributed to glioma's invasion and angiogenesis, and its expression related to poor outcome in IDH1 mutant patients [30]. Our findings indicate that chemokine signaling pathway plays a fundamental role in highly malignant IDH wild-type astrocytomas, and it can be considered as a potential therapeutic target for astrocytomas.

Taking these together, we discovered that calcium signaling pathway correlated with astrocytoma tumorigenesis and might be a potential biomarker for diagnosis or therapy, then we observed that IDH mutation led to the epigenetic and expression characteristics change in complement and coagulation cascades pathway. Finally, we preliminarily described a molecular classification of IDH wild-type astrocytoma in Chinese population, and the chemokine signaling pathway could be the biomarker.

## MATERIALS AND METHODS

### Study population

This project's protocol was approved by the Ethics Committee of Xiangya School of Medicine, Central South University with registration number of CTXY-1300041-3. 33 astrocytic tumor tissues and 9 non-tumor brain tissues were retrospectively obtained from Hunan Province Tumor Hospital and Xiangya Hospital of Central South University (Changsha, Hunan, China) between 2004 and 2011 with informed consent. And all brain tissues were from cerebral cortex. The clinical and pathological data were collected from all patients with histologically confirmed astrocytoma who underwent surgical resection.

### Data collection

Genomic DNA extracted from brain tissues was bisulfite converted using the Zymo EZ DNA Methylation Kit (Zymo Research) following the manufacturer's protocols. Genome-wide DNA methylation analysis was performed by Illumina 450K Infinium Methylation BeadChip, which quantitatively measures more than 485,000 methylation sites covering 99% of Ref-Seq genes. The IDAT files were input to R software by the Bioconductor minfi package to normalize arrays.

Total RNA was isolated from tissue specimens using the TRIzol Reagent (Invitrogen) and purified with NucleoSpin RNA clean-up (MACHEREY- NAGEL) according to the manufacturer's instructions. The quality and integrity of the RNA were verified by gel electrophoresis. The total microRNA was purified using mirVana miRNA Isolation Kit (Ambion) and quantified with NanoDrop ND-1000 spectrophotometer (NanoDrop). The microRNA expression profiling was performed using Human miRNA Microarray, Release 19.0, 8×60K (Agilent Technologies), which contains 2006 human miRNAs. 200 ng of total RNA was labelled and hybridized with miRNA Complete Labeling and Hyb Kit (Agilent Technologies) following the manufacturer's instructions. Slides were scanned by Agilent G2565CA Microarray Scanner and scanned images of microarray were processed with Agilent Feature Extraction Software v10.7 (Agilent Technologies).

mRNA and lncRNA expression were measured using LncRNA+mRNA Human Gene Expression Microarray V3.0, 4×180K, which contains 28,844 human mRNAs and 37,581 human lncRNAs. After labeling, hybridization and washing, slides were scanned with the Agilent G2565CA Microarray Scanner (Agilent Technologies). Raw data was extracted through Agilent Feature Extraction Software v10.7 (Agilent Technologies).

### Statistical analysis

The DNA methylation level for each probe is represented as a β-value ranging from 0 (completely unmethylated) to 1 (completely methylated) [β = intensity of the methylated allele (M) / (intensity of the unmethylated allele (U) + intensity of the methylated allele (M) + 100)]. Probes located on the X or Y chromosomes and probes containing SNPs or “NA” values were excluded prior to the data analyses.

Row data of miRNAs, mRNAs, and lncRNAs was quantile normalized and log 2-scale transformed in GeneSpring GX software (Agilent Technologies). For data quality control, probes located on the X or Y chromosomes and probes not reaching a detection rate of 60% in the upregulated group were excluded. After the filtering procedure, the normalized expression values of mRNA, lncRNA and miRNA probes were median centered, resulted in 17,995 genes, 25,416 lncRNAs and 628 miRNAs for the subsequent analysis respectively.

The two-sided unpaired Student's t-test was performed and the fold changes were calculated between tumor and non-tumor groups. Benjamini and Hochberg method was used to calculate false discovery rate (FDR). Adjusted P-value<0.05 and additionally fold change≥2 were the cut-off value of statistical significance.

### qRT-PCR of mRNAs, miRNAs, and lncRNAs

qRT-PCR was used to validate the expression of mRNAs, miRNAs, and lncRNAs. The total RNA was reverse transcribed to cDNA by PrimeScript RT-PCR Kit (Takara, Dalian, China) according to the manufacturer's protocol. qRT-PCR was performed in Roche LightCycler 480 II Real-Time PCR system (Roche Diagnostics Ltd., Rotkreuz, Switzerland), the relative expression level was calculated using 2^−ΔΔCt^ method. All primers used are listed in Supplementary Table 1.

### Pyrosequencing of IDH mutations

Pyrosequencing was used to detect the IDH mutation and validate the DNA methylation. Pyrosequencing was performed on PyroMark Q24 system with the Pyro Gold reagents kit according to the manufacturer's protocol, and the result data was analyzed using the PyroMark Q96 software. Detailed information of the pyrosequencing primers is provided in Supplementary Table 2.

### Quantitative microsatellite analysis of 1p/19q codeletion

Quantitative microsatellite analysis (QuMA) was performed using 4 microsatellite markers (D1S214, D1S468, D1S2736 and D1S2783) on chromosomes 1p and 3 microsatellite markers (D19S408, D19S596, and D19S867) on chromosomes 19q as described previously [31]. And five primer sets for the reference pool were chosen from chromosomes in unaltered gliomas: 3p (D3S1554), 5q (D5S643), 8q (D8S1800), 12q (D12S1699), and 21p (D21S1904) (Supplementary Table 3). The Taqman probe was 5’-Fam-TGTGTGTGTGTGTGTGTGTGT-Tamra-3’. According to the PCR cycle number (Ct) value, we calculated ΔCt (ΔCt=Ct [microsatellite]-Ct [reference]), ΔΔCt (ΔΔCt=ΔCt [tumor]-ΔCt [normal]), and the DNA relative copy number (DNA relative copy number=2×2^-ΔΔCt^). The SD and mean value of ΔCt [normal] for each microsatellite marker was used to create a tolerance interval (TI) with a confidence interval of 95%. TI=2±SD×2.420, where 2.420 is a two-sided tolerance limit factor for a total number of 70 measurements. Based on the TI, it is defined as a loss or a gain when copy number was less or more than TI, respectively.

### MGMT methylation

A two-probe model (probes cg12434587 and cg12981137) was used to determine MGMT methylation. M-value of each probe could be calculated by the equation: The equation for this model is given below:

$$\text{M }=\text{ log2}(\frac{Beta}{1\;-\;Beta})$$

logit(y) = 4.3215+0.5271*M (cg12434587)+ 0.9265*M (cg12981137)

The methylation probability y was computed using the inverse logit function, and the cut-off value was 0.358.

## SUPPLEMENTARY MATERIALS FIGURES AND TABLES


