# Supplementary material for: The molecular classification of astrocytic tumors
Source: Oncotarget. 2017 Oct 25;8(56):96340–50. doi: 10.18632/oncotarget.22047 (PMC5707104; doi:10.18632/oncotarget.22047)
Supplement: Supplementary file 1 [file oncotarget-08-96340-s001.pdf]

The molecular classification of astrocytic tumors

SUPPLEMENTARY MATERIALS

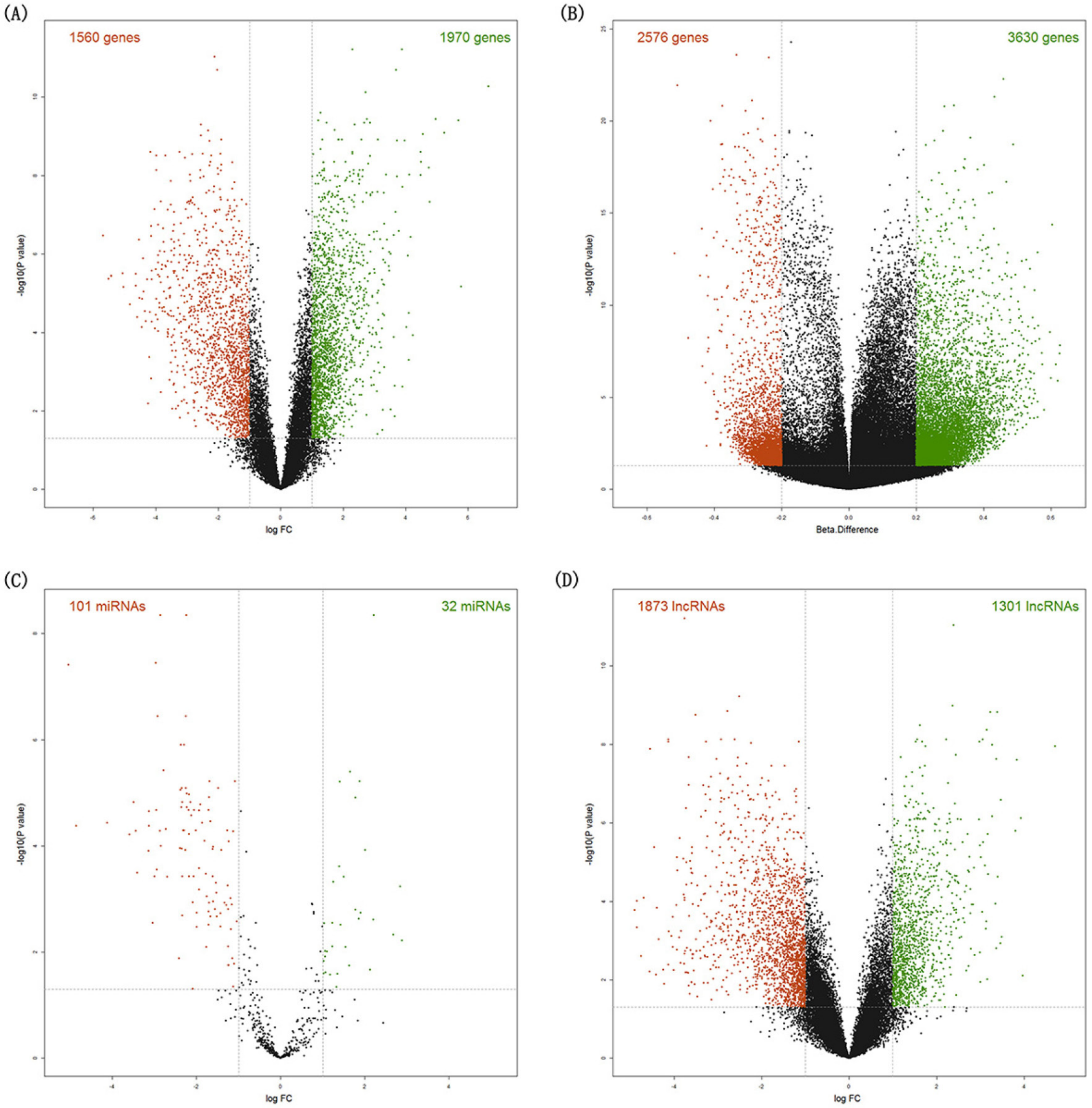

Supplementary Figure 1: Volcano plots of differential signatures analysis. X-axis: log2 fold change; Y-axis: -1×log10 (BH adjusted p-value) for each probes; Vertical dotted lines: fold change ≥2 or ≤2; Horizontal dotted line: the significance cutoff (BH adjusted p-value =0.05). **(A)** mRNA, **(B)** DNA methylation, **(C)** miRNA, **(D)** lncRNA.

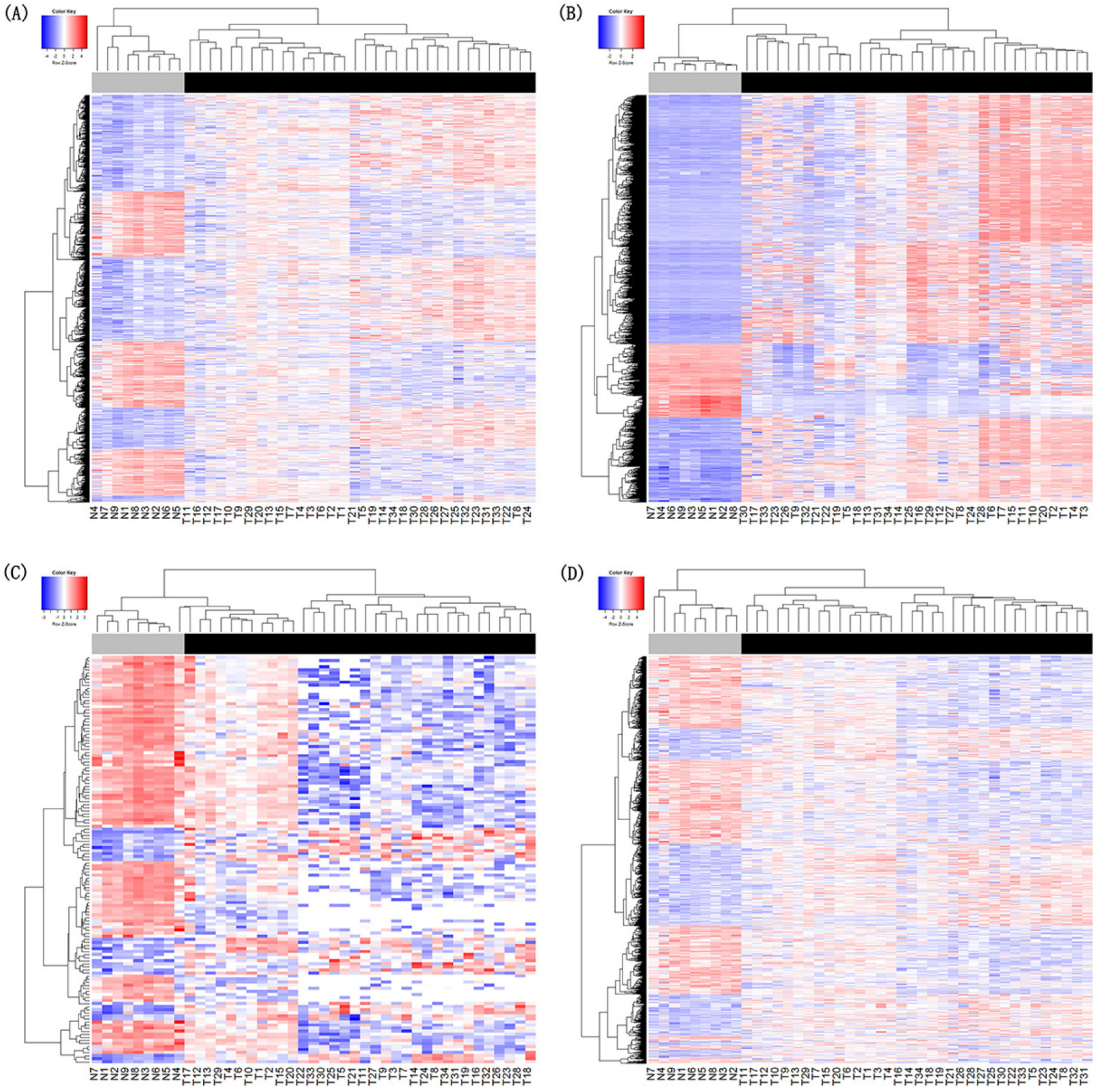

Supplementary Figure 2: Two-dimensional hierarchical clustering of the significantly differential signatures in all samples (33 tumor tissues in black vs. 9 NTL tissues in gray). Signatures are in rows; samples are in columns. **(A)** Two-dimensional hierarchical clustering was performed using 3627 differentially expressed genes between tumors and NTL tissues. **(B)** Two-dimensional hierarchical clustering was performed using the top 5000 differentially methylated probes between tumors and NTL tissues. **(C)** Two-dimensional hierarchical clustering was performed using 136 differentially expressed miRNAs between tumors and NTL tissues. **(D)** Two-dimensional hierarchical clustering was performed using 3334 differentially expressed lncRNAs between tumors and NTL tissues.

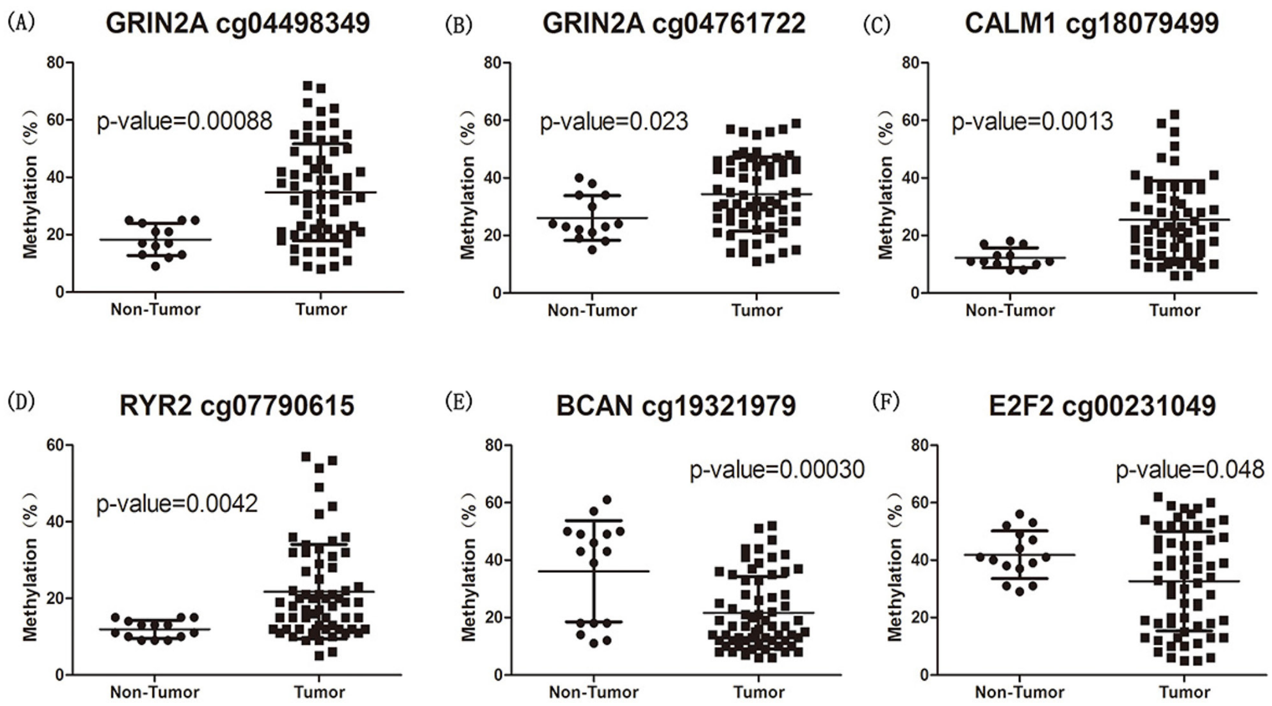

Supplementary Figure 3: Validation of the differentially methylated DNA methylation sites. The methylation level of 6 differentially methylated DNA methylation sites were validated by pyrosequencing in 65 astrocytic tumors and 16 non-tumor brain tissues. **(A)** GRIN2A cg04498349, **(B)** GRIN2A cg04761722, **(C)** CALM1 cg18079499, **(D)** RYR2 cg07790615, **(E)** BCAN cg19321979, **(F)** E2F2 cg00231049.

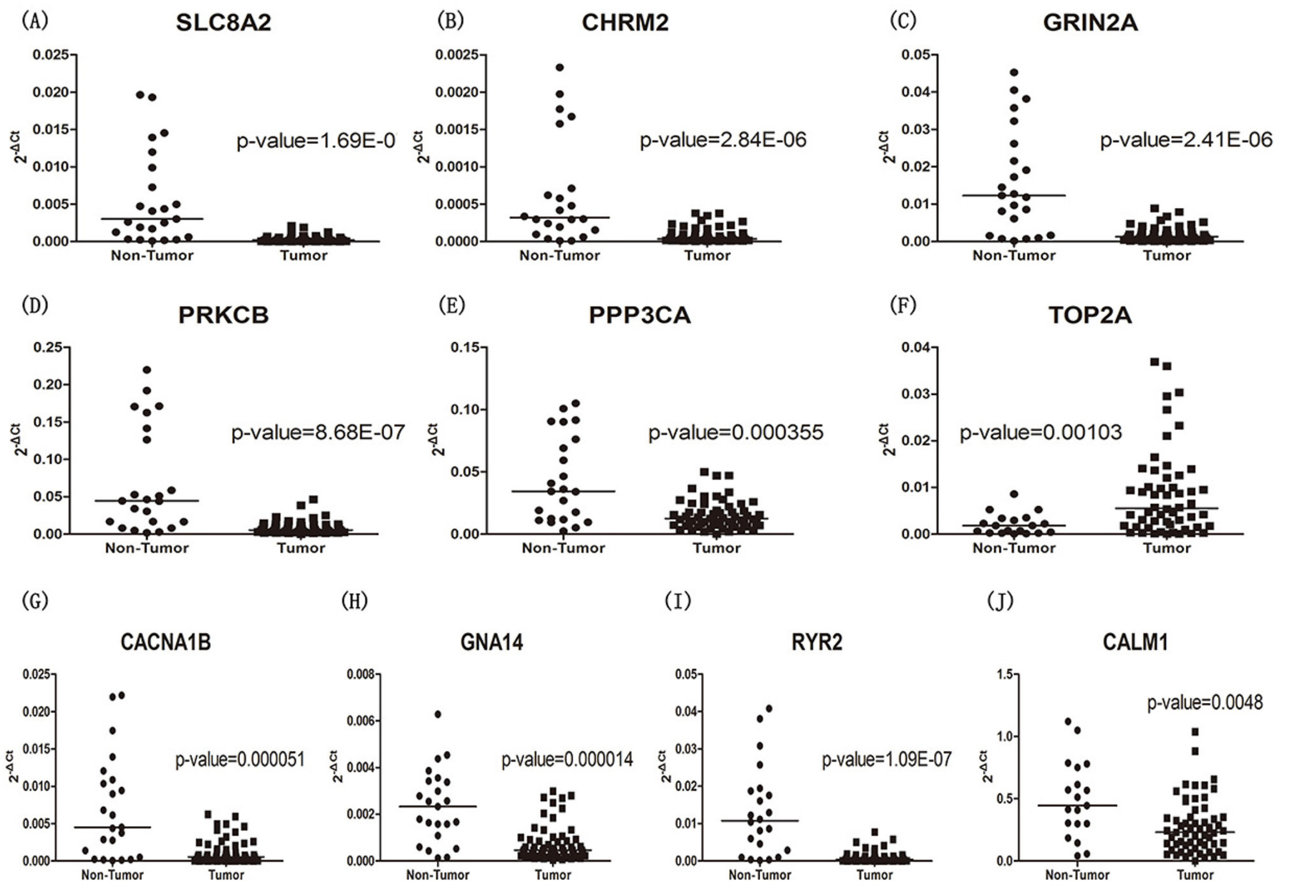

Supplementary Figure 4: Validation of the differentially expressed genes. The expression of 10 differentially expressed genes were validated by quantitative real-time PCR in 65 astrocytic tumors and 23 non-tumor brain tissues. **(A)** SLC8A2, **(B)** CHRM2, **(C)** GRIN2A, **(D)** PRKCB, **(E)** PPP3CA, (F) TOP2A, **(G)** CACNA1B, **(H)** GNA14, **(I)** RYR2, **(J)** CALM1.

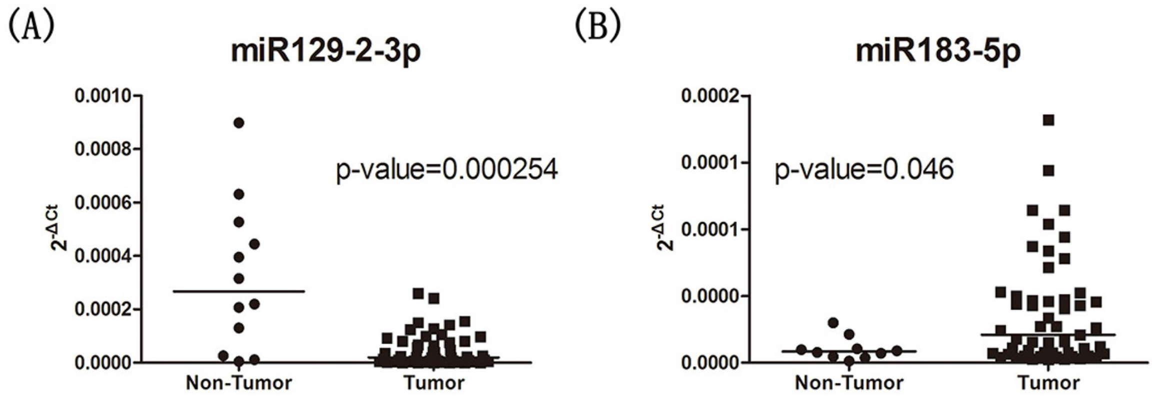

Supplementary Figure 5: Validation of the differentially expressed miRNAs. The expression of 2 differentially expressed miRNAs were validated by quantitative real-time PCR in 60 astrocytic tumors and 12 non-tumor brain tissues. **(A)** miR129-2-3p, **(B)** miR183-5p.

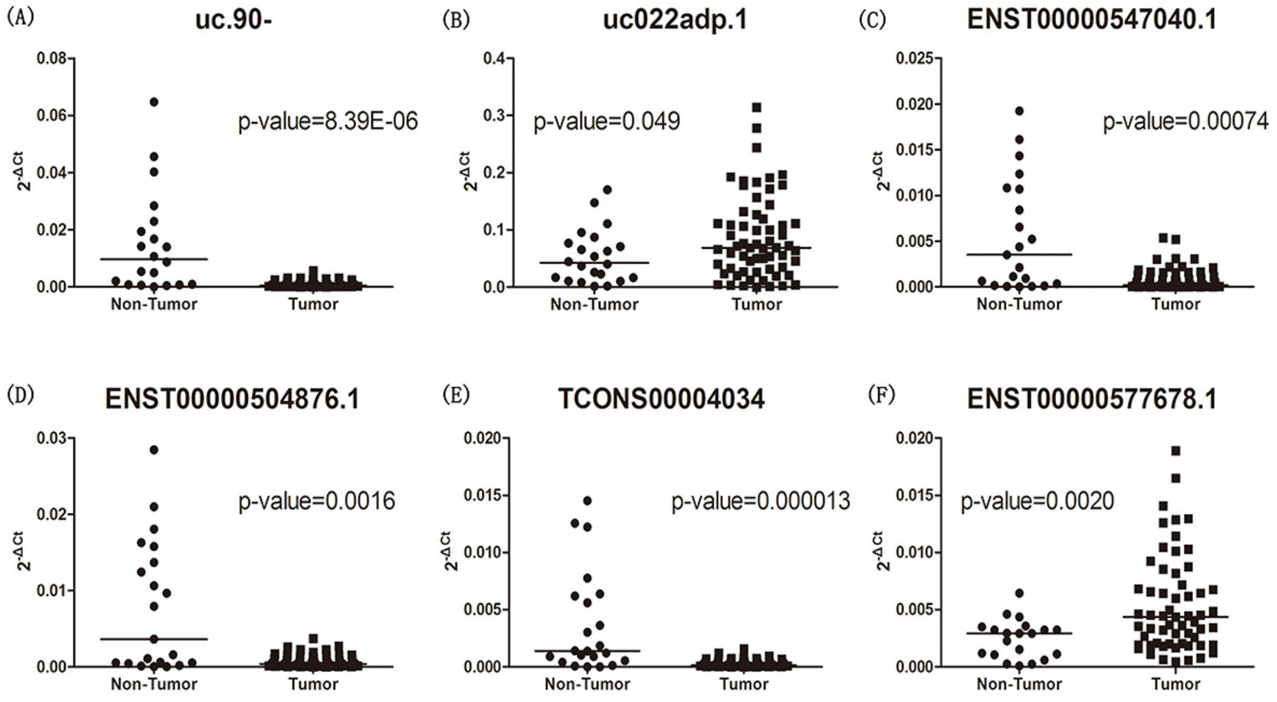

Supplementary Figure 6: Validation of the differentially expressed lncRNAs. The expression of 6 differentially expressed miRNAs were validated by quantitative real-time PCR in 65 astrocytic tumors and 23 non-tumor brain tissues. **(A)** uc.90-, **(B)** uc022adp.1, **(C)** ENST00000547040.1, **(D)** ENST00000504876.1, **(E)** TCONS00004034, **(F)** ENST00000577678.1.

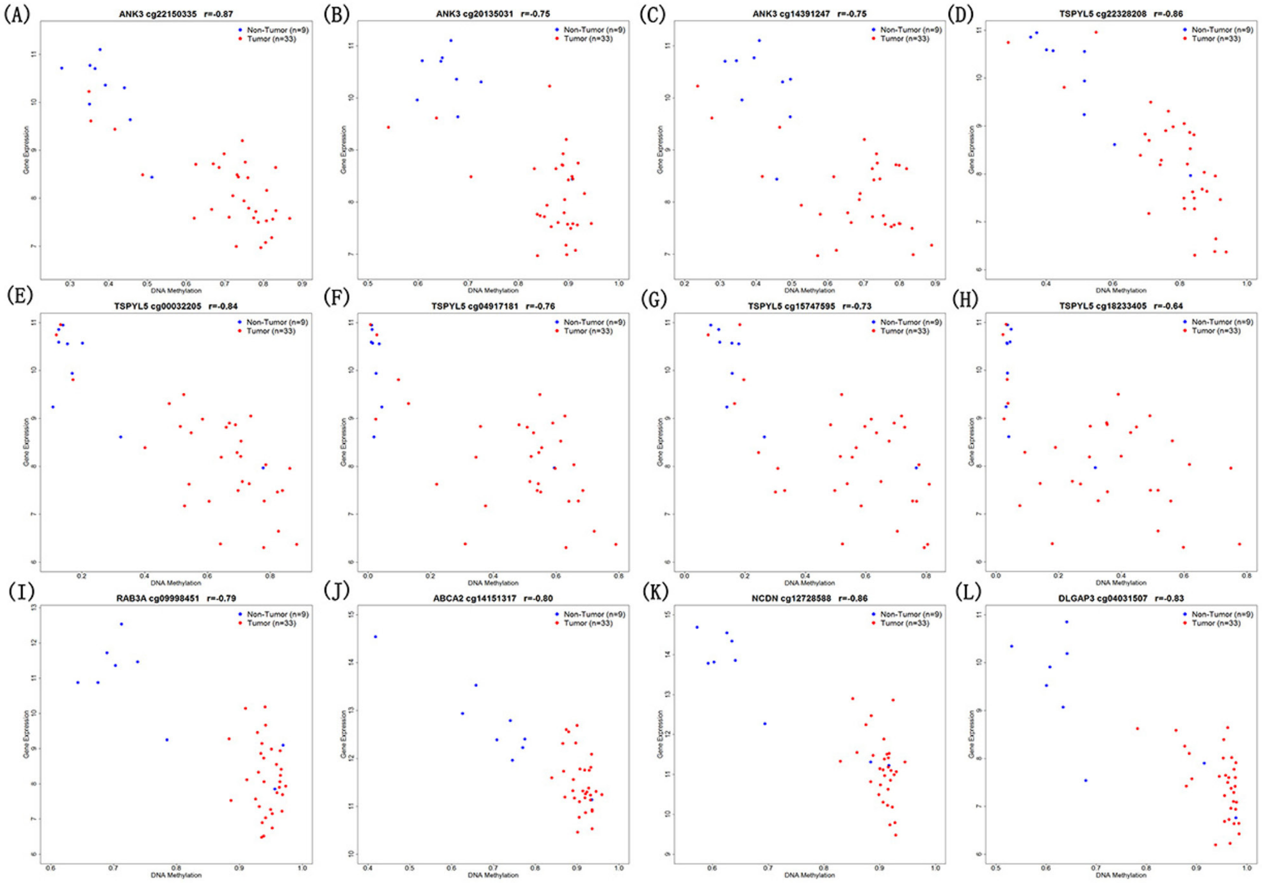

Supplementary Figure 7: Scatterplots of gene expression versus DNA methylation in tumors and non-tumor tissues for select genes. **(A-C)** ANK3, **(D-H)** TSPYL5, **(I)** RAB3A, **(J)** ABCA2, **(K)** NCDN, **(L)** DLGAP3.

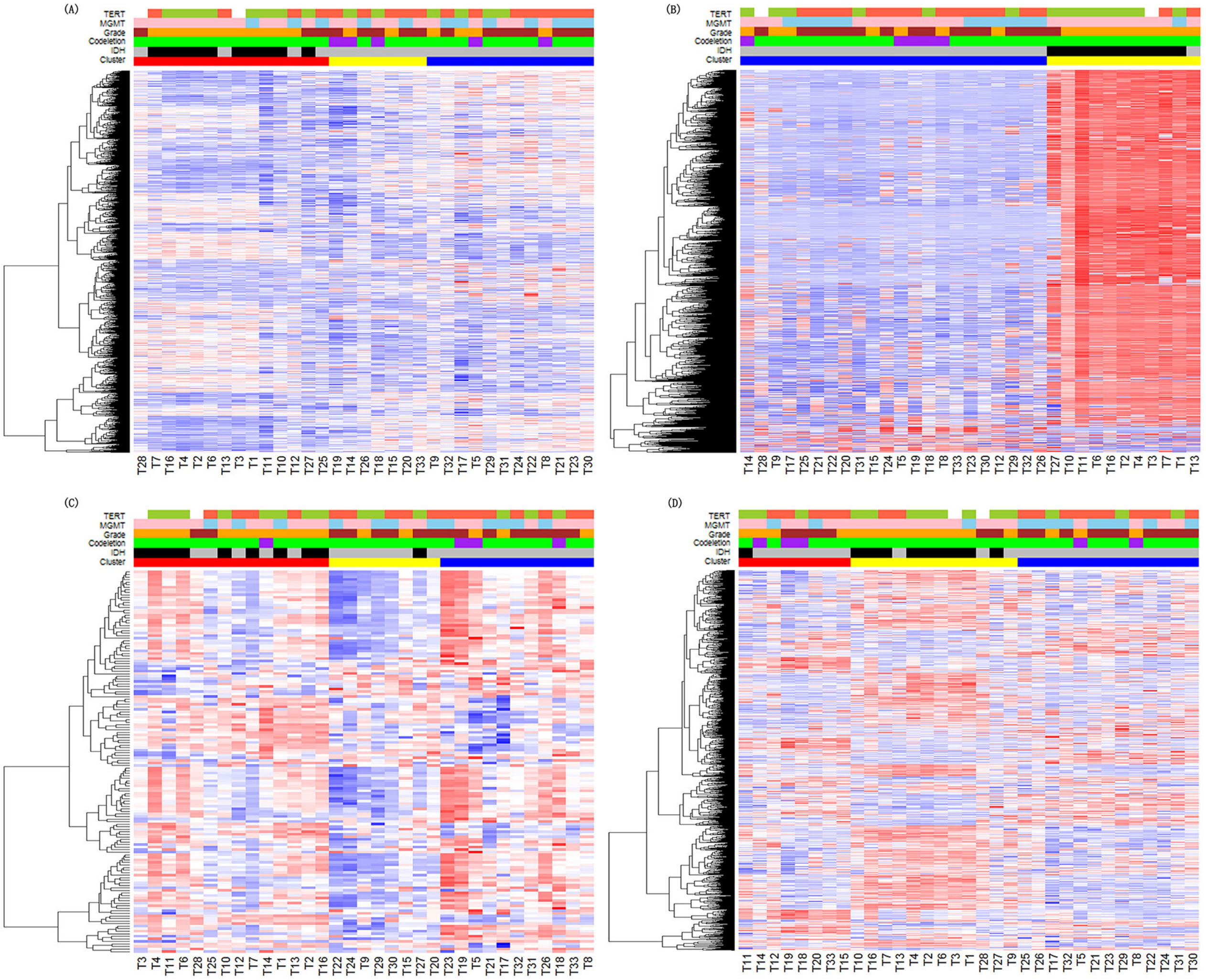

Supplementary Figure 8: Unsupervised hierarchical clustering analysis of DNA methylation and RNA expression on all astrocytomas. The color bars indicate various molecular features: black, IDH mutation (n=10); grey, IDH mutation (n=23); purple, 1p/19q codeletion (n=5); green, no 1p/19q codeletion (n=28); brown, GBMs (n=16); orange, lower-grade astrocytomas (n=17); pink, high MGMT promoter methylation (n=21); skyblue, low MGMT promoter methylation (n=12); tomato, TERT promoter mutation (n=17); yellowgreen, no TERT promoter mutation (n=14). White indicates missing value. **(A)** mRNA, cluster memberships are indicated by the color bar: red, Cluster m1 (n=14); yellow, Cluster m2 (n=7); blue, Cluster m3 (n=12). **(B)** DNA methylation, cluster memberships are indicated by the color bar: blue, Cluster M1 (n=22); yellow, Cluster M2 (n=11). **(C)** miRNA, cluster memberships are indicated by the color bar: red, Cluster mi1 (n=14); yellow, Cluster mi2 (n=8); blue, Cluster mi3 (n=11). **(D)** lncRNA, cluster memberships are indicated by the color bar: red, Cluster lnc1 (n=8); yellow, Cluster lnc2 (n=12); blue, Cluster lnc3 (n=13).

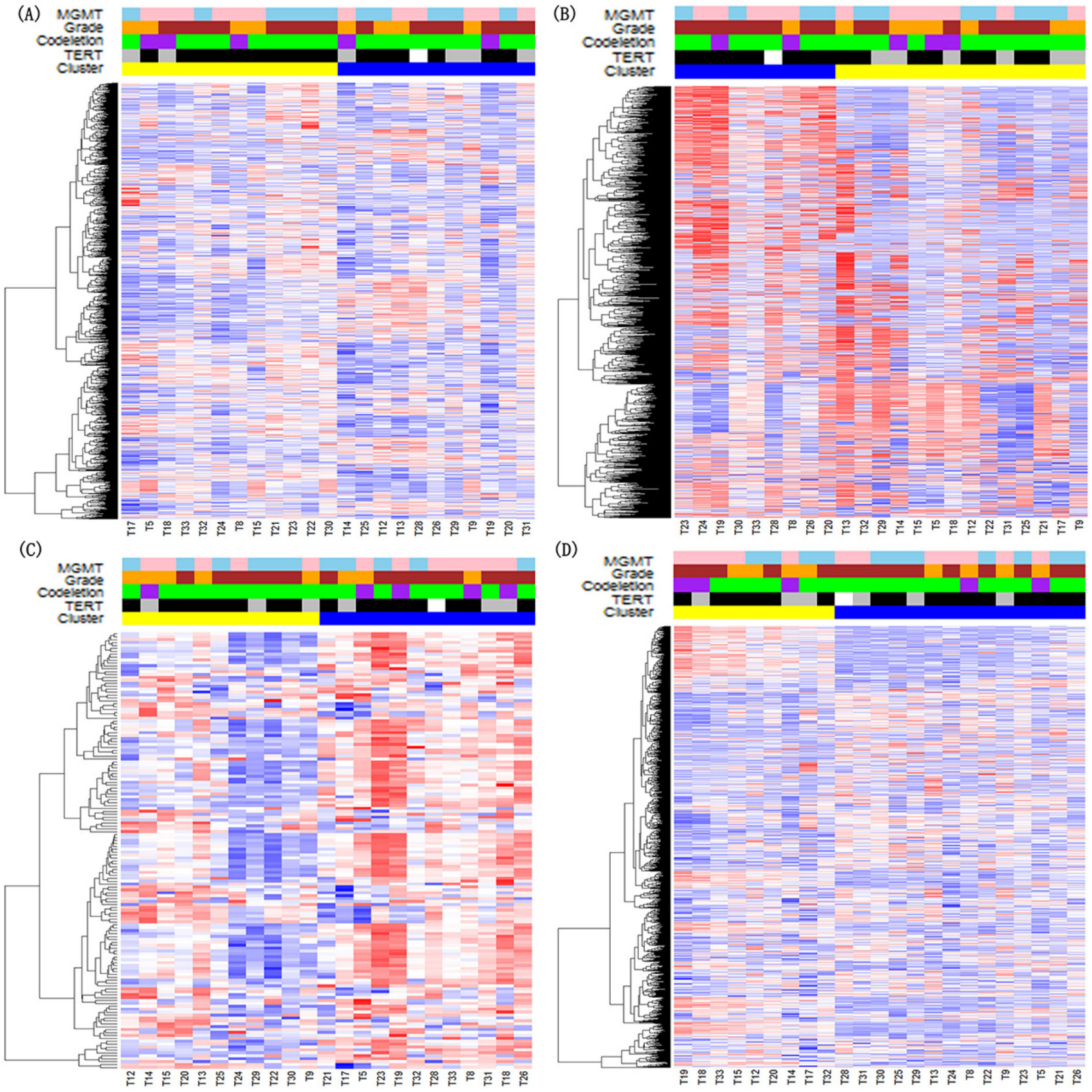

Supplementary Figure 9: Unsupervised hierarchical clustering analysis of DNA methylation and RNA expression on IDH wild-type astrocytomas. The color bars indicate various molecular features: black, TERT promoter mutation (n=16); grey, no TERT promoter mutation (n=6); purple, 1p/19q codeletion (n=5); green, no 1p/19q codeletion (n=18); brown, GBMs (n=15); orange, lower-grade astrocytomas (n=8); pink, high MGMT promoter methylation (n=12); skyblue, low MGMT promoter methylation (n=11). White indicates missing value. **(A)** mRNA, cluster memberships are indicated by the color bar: yellow, Cluster m1 (n=12); blue, Cluster m2 (n=11). **(B)** DNA methylation, cluster memberships are indicated by the color bar: blue, Cluster M1 (n=9); yellow, Cluster M2 (n=14). **(C)** miRNA, cluster memberships are indicated by the color bar: yellow, Cluster mi1 (n=11); blue, Cluster mi2 (n=12). **(D)** lncRNA, cluster memberships are indicated by the color bar: yellow, Cluster lnc1 (n=9); blue, Cluster lnc2 (n=14).

Supplementary Table 1: DNA sequences of the primers for qRT-PCR

Primer	Forward	Reverse
TOP2A	ACCATTGCAGCCTGTAAATGA	GGGCGGAGCAAAATATGTTCC
SLC8A2	CGGGCAGTGGTGTACTTTGT	TGAAACGGTCGGCGATG
PRKCB	AGCCCCACGTTTTGTGACC	GCTGGGAACATTCATCACGC
RYR2	CCGGAAACAGTATGAAGACCAGCTA	CACACAACGCTGGCAATTCAC
GRIN2A	TGGCCTCACCGGGTATGATT	CAATGCCGTCCCTCACTCTC
CALM1	TACTTCGTGTGCTCCGACCCAT	AGTCCACAGCCACAGCCTACTC
CACNA1B	GGTCTCTGGTGGTTTTGTTCT	AGGAGATTTCCGTTGTGTGG
CHRM2	CAAGGGAGAAAGAGAACCGGCA	ACCTGTCGCTGGTTTCGCTC
GNA14	TGACAACGAGAATCGCATGGA	GCTCTGACATCCTGTTTCGGT
PPP3CA	GATATTGATGCGCCAGTCAC	CCCTAAGAAGAGGTAGCGAGTG
ENST00000577678.1	CAGGCTGTATTACTGTCTTTGA	AACATGAACCTTATATTTTCGA
uc022adp.1	ATGGATTTGATGACTGGTATTAGA	CAAAACTTTCAAGGTTAAAACGTA
ENST00000547040.1	TTGTTTAAGCTTCGGGATTT	GCCCTGCATGGTATTATTTG
ENST00000504876.1	ATGAAATACTGCTGGCCTACC	ACTTTGGGTCCTTTTTTGTGT
TCONS_00004034	AACAACTTCTGGGGTTCAAATC	GCTGGGAGCAATAGGAGATAAT
uc.90-	TCCAAGACAGGAGAGAGTTT	TTACCAGGGGGATGACGAAT

Supplementary Table 2: DNA sequences of the primers for pyrosequencing

Primer	Forward	Reverse	Pyrosequencing
IDH1	Biotin-CATAATGTTGGCGTCAAATGTG	ACATGCAAAATCACATTATTGCC	TGACTTACTTGATCCCC
IDH2	GTTCAAGCTGAAGAAGATGTGG	Biotin-TGTGGCCTTGTACTGCAGAG	AAGCCCATCACCATT
CALM1 cg18079499	GTAGGGTTAGAGGGAATTAGTAGTA	Biotin-CTTTTCTCCTCCCAACTCATCTTTTAA	TGATAGAGGTTTGTTATTTGTTTTT
GRIN2A cg04498349	GGTTTTTGTAAGGTGAAGAGTGA	Biotin-TCCTACTACTAAATTCCATACCTAACT	AGGTGAAGAGTGAGA
GRIN2A cg04761722	GGTTTTTGTAAGGTGAAGAGTGA	Biotin-TCCTACTACTAAATTCCATACCTAACT	AGGTGAAGAGTGAGA
BCAN cg19321979	TTGGTATTTAGGGGAGGGAATTTAGGTA	Biotin-CTCCTTTCCCAAACTCTCTTTACTT	ATTTAGGTATTTGGTTGTTTT
E2F2 cg00231049	Biotin-GGGTGGTTTGTTGTAAGGAGAT	CTCCTATACCAAACACTACTTAACACTCT	ACACTACTTAACACTCTTC
RYR2 cg07790615	Biotin-GGTGTTGAGTAATTTTTATTTGGAGATT	CCCCCTAAAATCCCTAAACTCATTCCC	CCTAAACTAAACTAAAACACTA

Supplementary Table 3: DNA sequences of the primers for QuMA

Primer	Forward	Reverse
D1S214	CCGAATGACAAGGTGAGACT	AATGTTGTTTCCAAAGTGGC
D1S468	TAAAATATTAGGTCAAACCATG	ATGGCTGCATATAATGTTG
D1S2736	TACCTCCAGGGTATTCTTGG	TTTTTGAGGTGTGAGAGCAG
D1S2783	CCCTACCCTAATTCCACTG	GTTTATGTTTCACCTCCTATCC
D19S408	AGCTCTATGGGGTGGTGCC	GCCTCTTAGAGTTTTGGGAG
D19S596	CCACAGAGCAAGACTCGAT	GCCAGAGCCACTGTGT
D19S867	CAATGAAAATGCTTTGTAAAAC	CCTTCAGAGGTGACCAG
**Reference primers**		
D3S1554	ATTCATCTTGTTACTGTTCATTTGT	GGGCAAACCCAAAGACT
D5S643	TGGGCGACAGAGCCATC	TGTGGTGTGCCATTTATTGACT
D8S1800	CCATCAAATGTCGAACACTG	GTCCACCAATGCGTTAAAG
D12S1699	ACCTCATGCCTGTTAGG	TTCGTTCACATCCTGG
D21S1904	ATGAGTTCAGTGTTTCATGGACATC	AGCAAGATTACTGTCTGGTTTCCC

Supplementary Table 4: Genes in calcium signaling pathway

HUGO	HUGO gene name: function
ADRB3	Adrenoceptor Beta 3: G-protein coupled receptor activity and epinephrine binding
CACNA1B	Calcium Channel, Voltage-Dependent, N Type, Alpha 1B Subunit: calcium ion binding and voltage-gated calcium channel activity
CALM3	Calmodulin 3 (Phosphorylase Kinase, Delta): calcium ion binding and ion channel binding
CAMK2B	Calcium/Calmodulin-Dependent Protein Kinase II Beta: protein homodimerization activity and protein kinase activity
CAMK2G	Calcium/Calmodulin-Dependent Protein Kinase II Gamma: calmodulin binding and calcium-dependent protein serine/threonine phosphatase activity
CHRM2	Cholinergic Receptor, Muscarinic 2: G-protein coupled receptor activity and G-protein coupled acetylcholine receptor activity
EGFR	Epidermal Growth Factor Receptor: identical protein binding and chromatin binding
GRIN2A	Glutamate Receptor, Ionotropic, N-Methyl D-Aspartate 2A: calcium channel activity and N-methyl-D-aspartate selective glutamate receptor activity
HTR5A	5-Hydroxytryptamine (Serotonin) Receptor 5A, G Protein-Coupled: serotonin receptor activity
ITPKA	Inositol-Trisphosphate 3-Kinase A: calmodulin binding and calmodulin-dependent protein kinase activity
PLCB2	Phospholipase C, Beta 2: calcium ion binding and phosphoric diester hydrolase activity
PPP3CA	Protein Phosphatase 3, Catalytic Subunit, Alpha Isozyme: calcium ion binding and enzyme binding
RYR2	Ryanodine Receptor 2 (Cardiac): calcium ion binding and enzyme binding

Supplementary Table 5: The top significantly enriched KEGG pathways in integration analyses of IDH-mutated vs. IDH wild-type GBM

KEGG pathway	p-value	BH adjusted p-value
Cytokine-cytokine receptor interaction	2.663×10^{-7}	4.074×10^{-5}
Focal adhesion	1.390×10^{-5}	1.063×10^{-3}
ECM-receptor interaction	1.052×10^{-4}	5.351×10^{-3}
Complement and coagulation cascades	2.748×10^{-4}	0.010
Chemokine signaling pathway	2.294×10^{-3}	0.068
Regulation of actin cytoskeleton	9.932×10^{-3}	0.225
NOD-like receptor signaling pathway	0.023	0.401
Bladder cancer	0.043	0.567
Melanoma	0.044	0.538
O-Glycan biosynthesis	0.047	0.525
